# SARS-CoV-2 structural features may explain limited neutralizing-antibody responses

**DOI:** 10.1038/s41541-020-00264-6

**Published:** 2021-01-04

**Authors:** Martin F. Bachmann, Mona O. Mohsen, Lisha Zha, Monique Vogel, Daniel E. Speiser

**Affiliations:** 1 International Immunology Centre, Anhui Agricultural University, Hefei, China; 2 Department of Rheumatology, Immunology and Allergology, University Hospital Bern, Bern, Switzerland; 3 Department of BioMedical Research, University of Bern, Bern, Switzerland; 4 University Hospital and University of Lausanne, Lausanne, Switzerland

**Keywords:** Lymphocyte activation, Viral infection, Vaccines, Antibodies

## Abstract

Neutralizing antibody responses of SARS-CoV-2-infected patients may be low and of short duration. We propose here that coronaviruses employ a structural strategy to avoid strong and enduring antibody responses. Other viruses induce optimal and long-lived neutralizing antibody responses, thanks to 20 or more repetitive, rigid antigenic epitopes, spaced by 5–10 nm, present on the viral surface. Such arrays of repetitive and highly organized structures are recognized by the immune system as pathogen-associated structural patterns (PASPs), which are characteristic for pathogen surfaces. In contrast, coronaviruses are large particles with long spikes (S protein) embedded in a fluid membrane. Therefore, the neutralizing epitopes (which are on the S protein) are loosely “floating” and widely spaced by an average of about 25 nm. Consequently, recruitment of complement is poor and stimulation of B cells remains suboptimal, offering an explanation for the inefficient and short-lived neutralizing antibody responses.

The immune response to severe acute respiratory syndrome coronavirus 2 (SARS-CoV-2) infection is initiated by innate immune activation followed by antigen-specific B- and T-cell responses^1^. An important mechanism protecting from viral disease is the presence of virus-neutralizing antibodies, which is similar for almost all viruses causing acute disease followed by pathogen clearance. In line with this, all currently available anti-viral vaccines are primarily aiming at inducing virus-neutralizing antibodies. Neutralizing antibodies generally block binding of the virus to cellular receptors. In some cases, neutralizing antibodies may prevent conformational changes necessary for fusion of the virus with the cell membrane or proteolytic cleavage. Neutralizing antibodies against SARS-CoV-2 are directed against the spike (S) protein, which contains multiple antigenic epitopes in the receptor-binding domain (RBD) and non-RBD epitopes^2^. A major mechanism of neutralization is to block binding of RBD to angiotensin-converting enzyme 2 (ACE2), the cellular receptor for the virus. The RBD is localized at the tip of the S protein (Fig. 1). The receptor-binding motif (RBM) consists of about 70 aa within the RBD and represents the actual amino acids directly interacting with ACE2.

**Fig. 1:**
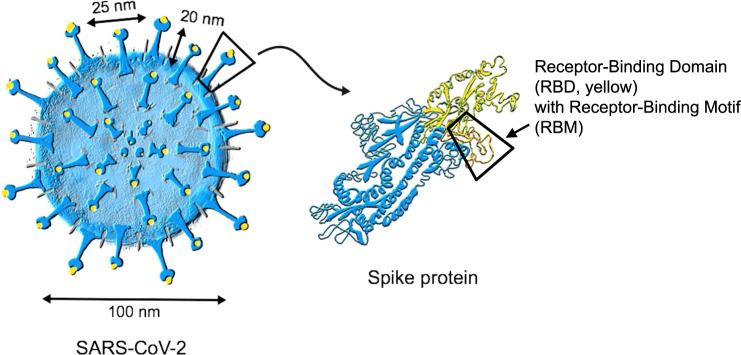
Structure of SARS-CoV-2. Coronaviruses have their names from the typical spikes which are made of the spike (S) protein that is inserted in the lipid bilayer membrane of the virus. The receptor-binding domain (RBD) and its receptor-binding motif (RBM) enable interaction with the cell surface receptor ACE2 mediating entry of the virus into host cells. This can be blocked by neutralizing antibodies. Therefore, most neutralizing epitopes are located on RBD/RBM. Besides the S protein, SARS-CoV-2 has two further viral surface proteins (not shown): envelope (E) and matrix (M).

Neutralizing antibodies are mostly directed against RBD and, in particular, RBM^3–5^. Indeed, RBD-specific antibodies closely correlate with neutralization in convalescent sera^3,6,7^. Although S is heavily glycosylated, RBD only shows little glycosylation (and one methylation) and the RBM is non-glycosylated, likely facilitating protein–protein interactions with ACE2. This may also indicate that glycosylation of S is probably not the reason for induction of poor neutralizing antibody responses. S is cleaved by furin and the serine proteases TMPRSS2 and TMPRSS4, enabling fusion of viral and cellular membranes, and consequent entry of viral RNA into the host cell^8^. This cleavage site may also be a target for neutralizing antibodies^9,10^. Overall, the RBD/RBM is the immunological Achilles heel of the virus. Therefore, the virus may have evolved strategies to mitigate induction of neutralizing antibodies against this domain.

## Duration and quality of neutralizing antibody responses to SARS-CoV-2

As for infections with other viruses, COVID-19 patients produce neutralizing antibodies at lower amounts than non-neutralizing ones. There is disagreement about the stability of neutralizing antibodies in COVID-19 patients, with several studies reporting stable persistence^11,12^, whereas others showing that neutralizing antibodies to coronaviruses wane relatively rapidly, or appear late and remain at low titers^13–15^. Some patients may even lack long-lasting antibodies^16^. Indeed, there is increasing evidence that protection from disease can be short-lived: some patients experienced COVID-19 twice within months, proven by a virus-free interval^17,18^.

Antibody titers generally show an early decay after infection, because the first antibody wave is based on short-lived plasma cells^19^. The second wave of antibodies is produced by more durable plasma cells^20^. Therefore, one cannot directly compare studies that differ in the time points at which antibodies were measured. In addition, studies differ with respect to laboratory methods. The “gold-standard,” i.e., neutralization assays that use live virus requires a safety level 3 laboratory, which is not always available. Although useful results are obtained by alternative approaches (pseudotype neutralizing assays or enzyme-linked immunosorbent assays designed to detect RBD-specific antibodies), they are less meaningful than the gold-standard^21^.

The disagreement about the duration of the neutralizing antibody response is not surprising, given the lack of long-term follow up as SARS-CoV-2 has appeared less than one year ago. Nevertheless, patients with minor or no symptoms often have only low and short-lived neutralizing antibody responses^13,14^. In addition, those patients are frequent in the current pandemic. Milder symptoms are observed when viral replication is restricted to the upper respiratory track. This occurs also in the usually mild common cold infections caused by the endemic seasonal coronaviruses, typically during winter and spring time^20^. For these viruses, antibody responses that protect from disease are short-lived^14^, in the range of 1 year^22^ or less^23^, and infections occur regularly, including re-infections with the same virus^15,22,24^.

Until last year, there existed only two coronaviruses that frequently cause severe disease, SARS-CoV-1 and Middle East Respiratory Syndrome Coronavirus (MERS-CoV). Neutralizing antibody responses to SARS-CoV-1 can be measured in most patients^25,26^ but may gradually disappear after recovery^27^. More is known about the MERS-CoV. This virus keeps on circulating in its natural hosts, the dromedary populations, and animals may experience re-infections. The camels have a high seroprevalence (>90%), but virus transmission is not blocked by previous infection^28^.

Different other reasons may hamper the immune response to coronaviruses, e.g., those concerning the innate immune system, which is key for early activation of inflammatory cells and cytokines. SARS-CoV-2 may inhibit dendritic cells^29^ and interferon (IFN)-I/III responses^30,31^. Relatively small percentages of patients with severe COVID-19 bear various genetic variants that compromise innate immune mechanisms^32^, in particular, type I IFN pathways^33^, or have autoantibodies against type I IFNs^34^, which are likely aggravating diseases severity. Regarding T cells, several studies showed impaired T-cell responses including CD4 helper and regulatory T cells^29,35^. This point could be important, as T cells may contribute to protection from disease, although this is not proven^36^. It is also necessary to state that for neutralizing antibodies, there is currently no proof that they indeed mediate protection from COVID-19. For most current vaccines, neutralizing antibodies are considered as correlate of protection from disease, although they do not necessarily equate to the only mechanism of protection. Finally, non-neutralizing antibodies such as those that fix complement on the viral surface or mediate antibody-dependent cellular cytotoxicity may also play a role, although this is not yet clear^36^.

In the following, we propose that structural adaption of the virus family is co-responsible for the inefficiency of neutralizing antibody responses to the S protein of SARS-CoV-2, in particular RBD/RBM.

## Structure function considerations for SARS-CoV-2

**Fig. 2:**
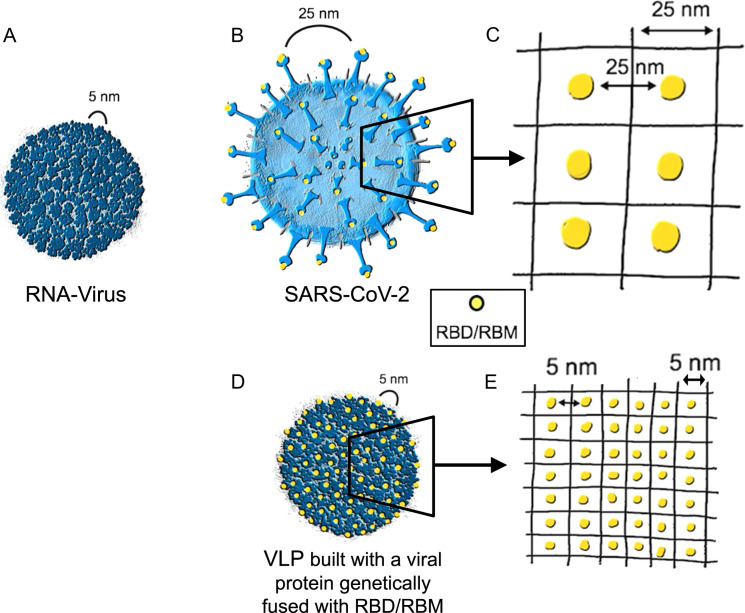
Distances between neutralizing epitopes. **A** An example of a classical RNA virus with a capsule made of multiple copies of only one protein that are rigidly structured, displaying highly immunogenic repetitive neutralizing epitopes spaced by 5–10 nm. This virus is built with 180 monomers and has a total viral diameter of 30 nm. **B**, **C** A coronavirus with its S proteins, showing the distance of the neutralizing epitopes of about 25 nm, which is large and unfavorable for triggering antibody responses. **D**, **E** A virus-like particle (VLP) built by a viral protein into which the RBM of SARS-CoV-2 is genetically inserted. This VLP displays the neutralizing epitopes with an optimized spacing of 5 nm.

Most viruses have highly organized, repetitive and rigid surfaces^37^. Typical RNA viruses cannot build up complex surfaces because of their limited genome of around 10,000 nucleotides (10 kb). Their capsid usually consists of multiple copies of only one or two proteins, often arranged in icosahedral symmetry^38^, readily and efficiently inducing neutralizing antibody responses^39^. As the vertebrate body is by and large devoid of such extracellular repetitive and organized structures, the immune system has evolved to recognize such antigen organization as a pathogen-associated structural pattern (PASP)^40^. In the 1970s, it was found that optimal immune responses are induced by at least 12–16 epitopes spaced by 5–10 nm, called the immunon^41^. Figure 2A shows a typical RNA virus, with a diameter of 30 nm and 180 copies of a single coat protein spaced by about 5 nm. Such viral particles efficiently cross-link B-cell receptors^42,43^ and are recognized by natural IgM, which induces the classical pathway of complement activation. This facilitates binding of viral particles to complement receptors followed by B-cell-mediated deposition on follicular dendritic cells causing efficient germinal center formation^44^. Furthermore, complement-dependent stimulation of CD21 on B cells facilitates induction of long-lived plasma cells, which is essential for durable antibody responses^45^. There is a vast literature confirming these considerations for human vaccines, where repetitiveness is important for inducing long-lived antibody responses^37,46^. Hence, repetitive, rigid structures spaced by 5–10 nm are optimal for complement and B-cell activation, resulting in durable antibody responses.

Figure 2A outlines the structure of SARS-CoV-2. It is immediately evident that the structure of this coronavirus is quite different. The virion has a relatively large body with a diameter of 100 nm (rather than 30–50 nm). Importantly, the S protein, which has a length of about 20 nm, is present rather scarcely, floating in a sea of lipid bilayer. As mentioned above, RBD is sitting at the top of S; therefore, some 70 nm away from the center. The viral surface area, in which RBD is moving within a two-dimensional space, 20 nm away from the lipid bilayer, can be calculated as 4 × *πr*^2^ = 4 × *π*70 nm^2^ = ca 62,000 nm^2^. Assuming an average number of ca. 100 S per virion, each S covers a surface area of about 620 nm^2^. This leads to a grid-length of 25 nm, which indicates that S is spaced by an average of 25 nm (Fig. 2C) rather than the 5–10 nm needed for optimal B-cell responses. Epitopes spaced by this large distance in a non-rigid manner are inefficient in cross-linking B-cell receptors or recruiting natural IgM antibodies, required for complement activation and induction of long-live plasma cells. Hence, SARS-CoV-2 dilutes it’s Achilles heel, RBD on S, in a sea of lipids and other proteins, avoiding potent neutralizing antibody responses.

The S protein forms a trimer. Consequently, the RBD will display three identical epitopes favorably spaced by about 3–5 nm. As discussed above, three epitopes are, however, not enough to optimally activate B cells. On the contrary, epitopes occurring in low numbers inhibit, rather than activate, B-cell responses. Indeed, Dintzis et al.^47^ concluded that increasing epitope density in a molecular structure increases its immunogenicity if the threshold number of ∼20 is reached. In contrast, increasing the density in a molecular structure below the threshold number increases its tolerogenicity^47^. Thus, trimeric RBD may reduce rather than increase neutralizing antibody responses.

An additional consideration is the length of the viral genome, as SARS-CoV-2 is encoded by 30,000 RNA nucleotides rather than the usually about 10,000 nucleotides seen for most other RNA viruses. Indeed, the longer genome of coronaviruses includes an RNA proofreading system, required for keeping the viral population viable based on sufficient genome stability^48^. Hence, in contrast to other RNA viruses, coronaviruses can build up relatively complex surfaces that allow evasion from immune recognition as a PASP and induction of enduring neutralizing antibody responses.

Besides the non-enveloped viruses, also many enveloped viruses (Influenza, Rabies, Sindbis, and Vesicular Stomatitis Virus) display highly immunogenic antigen arrays. Lytic viruses generally induce potent antibody responses and produce serotypes^37^. A notable exception were adenoviruses, which, similar to SARS-CoV-2, are lytic but also do not form serotypes. Parallel to coronaviruses, adenoviruses also dilute out the neutralizing epitopes on the surface of the virion thereby apparently avoiding stringent long-term neutralization (hence, no serotype formation). Similar to coronaviruses, adenoviruses have a proofreading replication system, as they are DNA viruses^49^. Hence, the presence of proofreading may allow viruses to escape immune recognition as a PASP.

We have previously discussed^37^ that viral structure predicts host antibody responses and serotype formation. An interesting observation was that viruses with highly organized and rigid surfaces induce T-cell independent antibody responses and form serotypes, whereas viruses with a less rigid structure avoid potent antibody responses and do not form serotypes. However, the “dilution” of the neutralizing epitopes performed by coronaviruses, as described in this study, is a unique strategy in the world of RNA viruses.

## Implications for vaccine design

If the inefficient and short-lived neutralizing antibody responses induced by SARS-CoV-2 are indeed caused by the unusually large distance between neutralizing epitopes embedded in a fluid membrane, this has important implications for vaccine design. Specifically, by simple genetic extraction of RBD or RBM from SARS-CoV-2 followed by grafting onto highly repetitive and immunogenic nanoparticles or virus-like particles (VLPs), one may render the poorly immunogenic RBD/RBM into a highly immunogenic version of it (Fig. 2D), with high numbers of accessible epitopes at optimal distancing (Fig. 2E). Indeed, chemical coupling or conjugation by the Spy-Catcher or similar methods of RBD to VLPs results in highly immunogenic vaccine candidates that stimulate production of high levels of neutralizing antibodies in test animals^50,51^. An alternative strategy, which facilitates large scale production, is represented by genetic fusion of RBD/RBM onto VLP-surfaces. Such approaches may represent attractive options that we and others are currently following^50,52,53^.

## Conclusion

SARS-CoV-2 induces inefficient neutralizing antibody responses that are short-lived. In contrast to the other RNA virus families, which display arrays of neutralizing epitopes spaced by 5–10 nm in a rigid manner, SARS-CoV-2 displays a low number of neutralizing epitopes spaced by 25 nm in a non-rigid manner, as the S protein is embedded in a fluid membrane. Hence, SARS-CoV-2 escapes an efficient neutralizing antibody response by structurally avoiding immunogenic display of its neutralizing epitopes.
